# Neurofilament Levels Are Reflecting the Loss of Presynaptic Dopamine Receptors in Movement Disorders

**DOI:** 10.3389/fnins.2021.690013

**Published:** 2021-12-03

**Authors:** Elena Diekämper, Britta Brix, Winfried Stöcker, Stefan Vielhaber, Imke Galazky, Michael C. Kreissl, Philipp Genseke, Emrah Düzel, Péter Körtvelyessy

**Affiliations:** ^1^German Center for Neurodegenerative Diseases (DZNE), Magdeburg, Germany; ^2^Institute for Experimental Immunology, EUROIMMUN Medizinische Labordiagnostika AG, Lübeck, Germany; ^3^Clinical-Immunological Laboratory Prof. Dr. Stöcker, Lübeck, Germany; ^4^Department of Neurology, University Hospital Magdeburg, Otto-von Guericke University, Magdeburg, Germany; ^5^Department of Nuclear Medicine, University Hospital Magdeburg, Otto-von Guericke University, Magdeburg, Germany; ^6^Institute for Cognitive Neurology and Dementia Research, Magdeburg, Germany; ^7^Department of Neuropathology, Charité—Universitätsmedizin Berlin, Corporate Member of Freie Universität Berlin and Humboldt-Universität zu Berlin, Berlin, Germany; ^8^Department of Neurology, Charité—Universitätsmedizin Berlin, Corporate Member of Freie Universität Berlin and Humboldt-Universität zu Berlin, Berlin, Germany

**Keywords:** neurofilament light chain, movement disorders, DaTscan, Parkinson’s Disease, CSF, alpha-synuclein, progranulin, neurofilament heavy chain

## Abstract

**Aims:** Neurofilament light chain (NfL) and phosphorylated neurofilament heavy chain (pNfH) are biomarkers for neuroaxonal damage. We assessed whether NfL and other biomarker levels in the CSF are correlated to the loss of presynaptic dopamine transporters in neurons as detected with dopamine transporter SPECT (DaTscan).

**Methods:** We retrospectively identified 47 patients (17 Alzheimer’s dementia, 10 idiopathic Parkinson’s disease, 7 Lewy body dementia, 13 progressive supranuclear palsy or corticobasal degeneration) who received a DaTscan and a lumbar puncture. DaTscan imaging was performed according to current guidelines, and z-scores indicating the decrease in uptake were software based calculated for the nucleus caudatus and putamen. The CSF biomarkers progranulin, total-tau, alpha-synuclein, NfL, and pNfH were correlated with the z-scores.

**Results:** DaTscan results in AD patients did not correlate with any biomarker. Subsuming every movement disorder with nigrostriatal neurodegeneration resulted in a strong correlation between putamen/nucleus caudatus and NfL (nucleus caudatus right *p* < 0.01, putamen right *p* < 0.05, left *p* < 0.05) and between pNfH and putamen (right *p* < 0.05; left *p* < 0.042). Subdividing in disease cohorts did not reveal significant correlations. Progranulin, alpha-synuclein, and total-tau did not correlate with DaTscan results.

**Conclusion:** We show a strong correlation of NfL and pNfH with pathological changes in presynaptic dopamine transporter density in the putamen concomitant to nigrostriatal degeneration. This correlation might explain the reported correlation of impaired motor functions in PD and NfL as seen before, despite the pathological heterogeneity of these diseases.

## Introduction

Currently the diagnosis of a movement disorder (MD) is based on clinical symptoms (Balestrino and Schapira, 2020). It is supported by imaging of the presynaptic dopamine transporter density with semi-quantitative determination in single photon emission computed tomography (SPECT) (Bajaj et al., 2013). Movement disorders are histopathologically heterogeneous entities with the idiopathic Parkinson’s disease (PD) being the most common form. It involves the degeneration of dopaminergic neurons in the pars compacta of the substantia nigra, which inhibits the motor-inhibiting part of the striatum resulting in a nigro-striatal degeneration (Balestrino and Schapira, 2020). Also, the putamen is affected early in the disease course (Kordower et al., 2013). There are a number of other parkinsonian-like neurodegenerative diseases associated with nigrostriatal degeneration called atypical parkinsonism or parkinsonian plus syndromes including diseases such as multiple system atrophy (MSA), progressive supranuclear palsy (PSP), corticobasal degeneration (CBD). Dementia with Lewy bodies (LBD) is also associated with PD but, in contrast, starts with mnestic syndromes and fads into PD with the mnestic syndromes being predominant throughout the disease (Cummings et al., 2011). There are numerous biomarkers established mirroring general, neuronal, or neuroaxonal neurodegeneration, but so far, no biomarker is known that specifically detects dopaminergic neurodegeneration nor proteinopathies caused by aggregated alpha-synuclein protein (Mollenhauer and Trenkwalder, 2009; Fayyad et al., 2019).

Dopaminergic neurodegeneration in the striatum can be visualized by dopamine transporter imaging. Nigrostriatal degeneration, in general, is associated with a decreased striatal presynaptic dopamine transporter density, which can be detected with SPECT using (123I) Ioflupane [iodine-123-fluoropropyl (FP)-carbo-methoxy-3 β-(4-iodophenyltropane) (CIT) FP-CIT as contrast agent (DaTscan) (Bajaj et al., 2013)]. DaTscan is an established *in vivo* imaging to diagnose suspected or unclear parkinsonian syndromes (Bajaj et al., 2013). Reduced density of dopamine transporters in the striatum is a suitable marker for nigrostriatal degeneration in PD or DLB. The binding pattern of the radioactive substance 123I correlates with the loss of presynaptic dopamine transporter detecting the radioactivity with SPECT indirectly mirroring the dopaminergic neurodegeneration in these neurons (Cummings et al., 2011). The neuronal fiber disintegrity in the striatum is already present in early stages of the disease (Bernheimer et al., 1973; Niznik et al., 1991; Duncan et al., 2013; Fazio et al., 2018), and therefore, DaTscan should be an early marker of neurodegeneration in these patients.

Alpha-synuclein (α-Syn) plays a major role in the pathogenesis of synucleinopathies, which include PD, LBD, and MSA. The presence of α-Syn in an aggregated form is a pathological hallmark in these diseases and may be responsible for neurodegeneration (Waxman and Giasson, 2008). In recent years, numerous biomarkers have been studied to support the clinical diagnosis of PD, LBD, and other diseases in cerebrospinal fluid (CSF) and serum (Mollenhauer and Trenkwalder, 2009; Parnetti et al., 2013; Van Dijk et al., 2013a,b; Hall et al., 2016; Majbour et al., 2016; Farotti et al., 2017). For alpha-synucleinopathies with movement disorders, some biomarkers indicating neurodegeneration, in general, show a reduced or increased level in patients with neurodegenerative movement disorders but lack a high specificity (Mollenhauer and Trenkwalder, 2009), although it has been shown that the combination of several biomarkers in CSF and serum can increase sensitivity and specificity for alpha-synucleinopathies (Parnetti et al., 2014; Majbour et al., 2016; Oosterveld et al., 2020).

Neurofilament light chain (NfL) is one of the four subunits of neurofilament proteins (Petzold, 2005; Oosterveld et al., 2020). This cytoskeletal protein is exclusively expressed in neurons and located particularly abundant in axons (Bridel et al., 2019). Neurofilaments in body fluids such as CSF are considered to be markers of neuronal and axonal injury (Bacioglu et al., 2016). Significantly elevated concentrations of NfL in CSF have been described in some neurological conditions, but no association with direct dopaminergic neurodegeneration has been described so far. However, the magnitude of increase shows a high degree of variability in clinically similar conditions. Parkinson’s disease must be differentiated from atypical parkinsonian syndromes as frontotemporal dementia from Alzheimer’s disease (AD) (Bridel et al., 2019). Human post-mortem brain studies have shown that NfL may be involved in Lewy body formation (Chu et al., 2012; Kordower et al., 2013; Moors et al., 2018). Another subtype of the neurofilament proteins is neurofilament heavy chain that can be abnormally phosphorylated (pNfH) in neurological disease. As a marker for axonal damage, pNfH gained attention as a diagnostic marker for neurological diseases such as amyotrophic lateral sclerosis (Gaiottino et al., 2013).

Progranulin (PGRN) is a protein with numerous functions in the brain involving lysosomal and microglial pathways (Kao et al., 2017). The involvement of PGRN in the PD pathomechanisms has been discussed lately (Tayebi et al., 2020). To our knowledge, there are no studies about CSF PGRN levels in patients with movement disorders. We added this precursor protein for granulin as a possible biomarker for neurodegeneration to the CSF biomarkers. Furthermore, we included total-tau (T-tau), which is associated with AD, PSP, and CBD, as a neuronal biomarker (Wang et al., 2013; Goedert et al., 2017).

As mentioned above, recent studies demonstrated that the diagnostic value may increase by combining biomarkers reflecting different pathological mechanisms in PD, such as axonal degeneration and α-Syn aggregation. For instance, CSF and serum NfL levels in combination with CSF α-Syn species achieve a high discriminative potential (Oosterveld et al., 2020). We aimed to assess whether these five CSF biomarkers could be linked to nigrostriatal degeneration as seen in the DaTscan in order to increase the significance of biomarkers indicating neurodegeneration in the dopaminergic system.

## Materials and Methods

This retrospective study is part of the Magdeburg Dementia Cohort Study approved by the Ethical Committee at the University Hospital Magdeburg, Germany (Approval Number 22/19). The study population included a cohort of 47 patients who received a lumbar puncture and presynaptic dopamine transporter imaging using SPECT. Every patient was diagnosed at the Department of Neurology, University Hospital Magdeburg, Germany between May 2012 and August 2017 according to the German clinical consensus criteria as proposed by the German Neurological Society for PD. We included patients with a nigrostriatal neurodegeneration (*n* = 30) suffering from PD (*n* = 10), LBD (*n* = 7), PSP (*n* = 12), and CBD (*n* = 1). Lewy body dementia, PSP, and CBD were diagnosed according to the international criteria at that time (Litvan et al., 1996; McKeith et al., 2005; Gilman et al., 2008; Armstrong et al., 2013) because of the small number of patients with PSP and CBD and because of the similar tau-mediated pathogenesis, we summarized these two clinical entities into one subcohort. Furthermore, a disease control with AD patients (*n* = 17) was used as a reference. In these 17 AD patients, DaTscan were performed as part of the clinical diagnostics due to unclear movement disorders and/or adjacent mnestic symptoms to differentiate, e.g., AD from LBD. Diagnosis of AD was made due to clinical course, neuropsychological data, and CSF biomarkers showing at least a pathologically affected amyloid metabolism (Jack et al., 2018). We used an age-matched control group (*n* = 13, mean age 64.1 ± 9.68; 52.4% female, 47.6% male) from Magdeburg, Germany, to compare the NfL levels with patients suffering from other neurological diseases without deviations in CSF. We used controls with non-neurological patients from the CSF laboratory at the department of Neurology, Magdeburg for PGRN (mean age = 69.87 ±, mean level = 0.77 ± 0.13 ng/ml). The NfL and PGRN control cohorts are published elsewhere (Körtvélyessy et al., 2015; Körtvelyessy et al., 2018). We designed a new non-immunological and non-neurodegenerative control cohorts for alpha-synuclein (mean age = 60.29 ± 8.8 years, mean level = 2,218.31 ± 888.19 pg/ml). For total-tau and pNfH levels, cutoffs according to the recommendation of the manufacturers were used (De Schaepdryver et al., 2018).

The dopamine transporter imaging was performed at the Department of Nuclear Medicine of the University Hospital Magdeburg (Otto-von Guericke University, Magdeburg Germany). SPECT imaging was performed 3 h after intravenous injection of 123-I-FP-CIT [180 MBq (± 2 Mbq); GE Medical]. An E.CAM (Siemens) with a fan-beam collimator (128 × 128 matrix; SPECT; 60 steps; 40 s/steps) was used. The relative uptake in the striatum was semi-quantified by using the three-dimensional automated functional brain analysis software BRASS^TM^ (Hermes BRASS software, Hermes Medical Solutions, Sweden). These semi-quantitative results are expressed in z-scores, which indicate the decrease in uptake of Ioflupane compared with a normal collective. The z-scores were calculated separately for the nucleus caudatus and putamen of each side.

The lumbar puncture was performed at the Department of Neurology at the University Hospital Magdeburg, Germany. The biomarker NfL (measured with an ELISA from Umandiagnostics, Sweden), PGRN (measured with an ELISA from Mediagnost, Germany), and T-tau (measured with an ELISA from Fujirebio, Belgium) were all measured at the Department of Neurology, Magdeburg, Germany, with PGRN and T-tau measured prospectively and NfL retrospectively. Due to the retrospective design of this study, NfL levels could not be measured in every patient. Alpha-synuclein and pNfH were measured in a batch in the EUROIMMUN laboratory in Lübeck, Germany.

Biomarker levels with log10 transformations were used to fit them to standard distribution.

Every statistical analysis was performed using Jasp 0.14 (University of Amsterdam, 2020). We correlated the CSF biomarker levels of PGRN, T-tau, α-Syn, pNfH, and NfL with z-scores obtained from the DaTscan for each brain region and performed a Pearson’s correlation once for all subgroups and once for combined groups. We used paired *t*-test to compare biomarker levels within cohorts and controls. Box plots were made with StatMacPlus V7.3.3.0 (AnalystSoft, United States).

## Results

### Epidemiology

Forty-seven patients received a dopamine transporter imaging *via* DaTscan and a spinal tap fulfilling the inclusion criteria. The mean age of all included patients was 69.7 (± 7.7) years, and 19 (40.4%) patients were female, 28 (59.6%) were male. We divided the patients into two cohorts (AD and MD) and subcohorts according to clinical characteristics. The highest mean age was observed in the subcohort LBD, the lowest in PSP and CBD. The mean age of all subcohorts did not differ significantly. Demographic characteristics for the cohorts and subgroups are presented in Table 1.

**TABLE 1:** Epidemiology data on patients.

**Characteristic^a^**	**AD (*n* = 17/X = 9)^e^**	**MD (*n* = 30/X = 13)^e^**	**PD (*n* = 10/X = 5)^e^**	**LBD (*n* = 7/X = 2)^e^**	**PSP + CBD (*n* = 13/X = 6)^e^**
Age (years)	70.2 ± 9.1	69.5 ± 6.9	71.6 ± 6.4	72.1 ± 7.6	66.5 ± 6.2
Gender					
No. (%) male	12 (70.6)	16 (53.3)	6 (60)	2 (28.6)	8 (61.5)
No. (%) female	5 (29.4)	14 (46.7)	4 (40)	5 (71.4)	5 (38.5)
NfL, pg/ml	1,859 ± 468.6^d^	2,041.77 ± 749.16^c^	1,301 ± 144.3^f^	2,107 ± 289.9^b^	2,637.3 ± 710^f^^,^^d^
pNfH, ng/ml	0.41 ± 0.18	0.50 ± 0.55	0.51 ± 0.39	0.45 ± 0.17	0.61 ± 0.76
PGRN, pg/ml	0.89 ± 0.18	0.87 ± 0.25	0.86 ± 0.22	0.81 ± 0.24	0.91 ± 0.29
T-tau, pg/ml	513.4 ± 240.8	301.6 ± 240.3^f^	247.8 ± 168.3^f^	319.00 ± 152.9^f^	333.6 ± 320.8
α-Syn, pg/ml	2012.9 ± 449.1	2,035.6 ± 1,043.9	1,885.2 ± 626.6	2,587.2 ± 1339.2	1,854.2 ± 1,100.3
Z Putamen right	1.79 ± 1.65	3.79 ± 1.45^h^	3.37 ± 1.56^f^	3.59 ± 1.12^f^	4.20 ± 1.59^h^
Z Nucleus caudatus right	2.04 ± 1.74	3.50 ± 1.38^g^	2.75 ± 1.43	3.51 ± 0.88^f^	4.01 ± 1.40^g^
Z Putamen left	1.75 ± 1.68	3.69 ± 1.78^h^	3.20 ± 1.86	3.02 ± 1.69	4.40 ± 1.65^h^
Z Nucleus caudatus left	1.99 ± 1.34	3.20 ± 1.64^f^	2.67 ± 1.60	2.93 ± 1.49	3.73 ± 1.71^g^

*Legends AD, Alzheimer’s disease; MD, movement disorders; PD, Parkinson’s disease; LBD, Lewy body dementia; PSP + CBD, progressive supranuclear palsy + corticobasal degeneration; NfL, neurofilament light chain; pNfH, phosphorylated neurofilament heavy chain; PGRN, progranulin; T-tau, Total-tau; α-Syn, alpha-synuclein.*

*^a^Data given as mean ± standard deviation.*

*^b^Compared with control group, p < 0.05.*

*^c^Compared with control group, p < 0.01.*

*^d^Compared with control group, p < 0.001.*

*^e^n, group sample size; X, number of NfL measurements.*

*^f^Compared with AD group, p < 0.05.*

*^g^Compared with AD group, p < 0.01.*

*^h^Compared with AD group, p < 0.001.*

### Dopamine Transporter Single Photon Emission Computed Tomography Are More Pathological in Movement Disorder Patients

Based on the software used and individual assessments at the division of nuclear medicine, 34 of 47 DaTscans were identified as pathological. In the AD cohort, 7 of 17 DaTscans revealed signs of pathological deviations, and in the MD cohort, 27 of 30 pathological results on the DaTscans have been seen. In patients with PD, 8 out of 10 scans were pathological, in patients with LBD, 7 out of 7 scans, and 11 out of 12 scans in patients with PSP and CBD. Only considering the quantitative results as z-scores, the lowest deviation from normal could be observed in patients with AD, the highest in patients with PSP and CBD (see Table 1).

### Neurofilament Light Chain Correlates Positively With Single Photon Emission Computed Tomography Pathology

**FIGURE 1:**
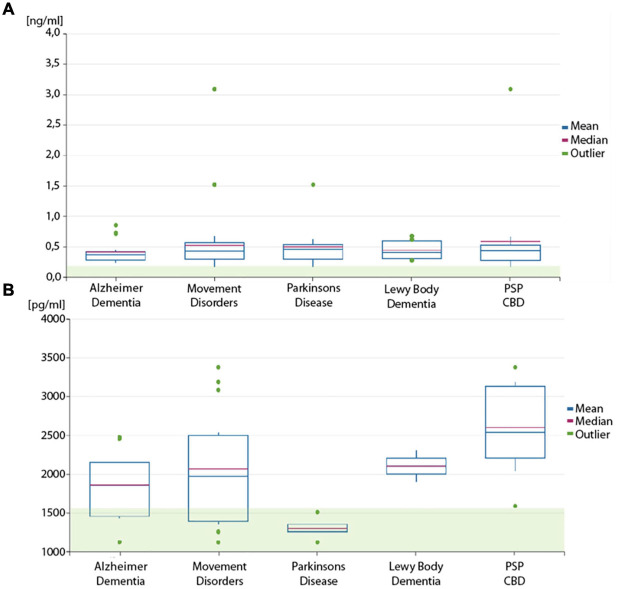
Box plot of neurofilament levels. Boxplots of **(A)** phosphorylated neurofilament heavy chain (pNfH) levels and **(B)** neurofilament light chain (NfL) in the cerebrospinal fluid (CSF) of the disease cohorts and subgroups. The transparent green box is indicating the normal range (mean + 1 standard deviation).

**FIGURE 2:**
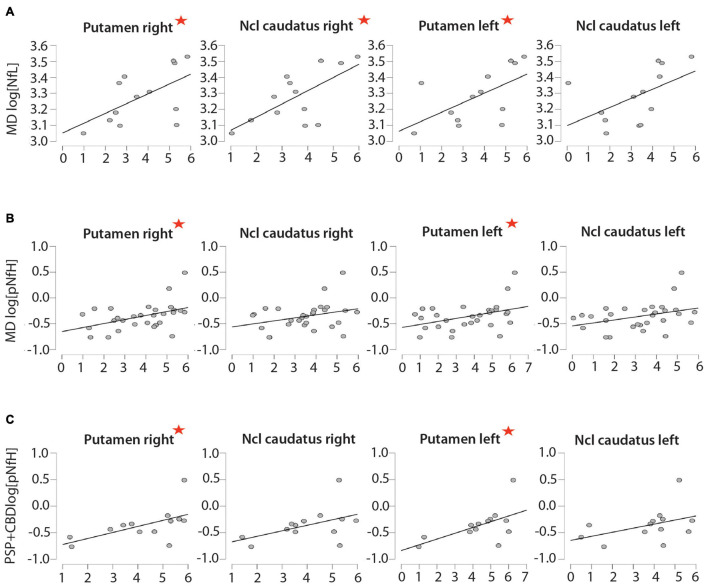
Correlations between neurofilament proteins and dopamine transporter single photon emission computed tomography (DaTscan). Correlations between CSF levels of **(A)** NfL and **(B,C)** pNfH and the results of DaTscan (Z-scores indicating the decrease in uptake compared with a normal collective) in patients with **(A,B)** movement disorders and **(C)** progressive supranuclear palsy + corticobasal degeneration (PSP + CBD). The red asterisk is indicating the significant difference. The *x*-axis shows the Z-scores of the DaTScan and on the *y*-axis the log (biomarker level).

When comparing NfL levels from MD and AD patients to the control group (mean 1,214.31 ± 448.1 pg/ml), we could find a significant increase in patients with AD (mean 1,859 ± 468.6 pg/ml; *p* < 0.001), MD (mean 2,041.77 ± 749.16 pg/ml; *p* < 0.01), LBD (mean 2,107 ± 289.9 pg/ml, *p* < 0.05), and PSP and CBD (mean 2,637.3 ± 710 pg/ml, *p* < 0.001) but not in patients with PD (mean 1,301 ± 144.3 pg/ml) (see Tables 1, 2 and Figure 1). In a second step, we analyzed the correlations between NfL levels and DaTscan results. No correlation between z-scores and CSF NfL levels could be found in the Alzheimer’s cohort (see Table 3 and Figure 2). Subsuming all movement disorders with nigrostriatal degeneration, positive correlations between NfL levels and nearly every striatal regions were found except the left nucleus caudatus showing a trend (*r* = 0.520, *p* = 0.069), (putamen right, *r* = 0.565, *p* < 0.05; nucleus caudatus right, *r* = 0.663, *p* < 0.05, and putamen left, *r* = 0.583, *p* < 0.05). No significant correlations between z-scores and CSF biomarkers could be observed for each of the MD subgroups.

**TABLE 2:** *t*-test of cerebrospinal fluid (CSF) biomarkers compared with non-neurodegenerative controls.

**α -Synuclein**		***P***	**Mean difference**	**Standard deviation**
	AD	0.384	205.464	233.355
	MD	0.487	182.753	261.231
	PD	0.287	333.152	307.744
	LBD	0.389	−368.918	422.246
	PSP + CBD	0.272	364.116	326.813

**Progranulin**				

	AD	0.038	−0.123	0.057
	MD	0.086	−0.100	0.057
	PD	0.265	−0.089	0.076
	LBD	0.580	−0.044	0.079
	PSP + CBD	0.130	−0.138	0.086

**NfL**				

	AD	0.004	−644.692	197.929
	MD	0.004	−827.462	252.907
	PD	0.545	−86.692	140.042
	LBD	0.019	−892.692	332.683
	PSP + CBD	< 0.001	−1423.026	265.794

*Significant results are marked in red.*

**TABLE 3:** Pearson’s correlation neurofilament light chain (NfL).

**Pearson’s correlation**	**AD**	**MD**	**PD**	**LBD**	**PSP + CBD**
Putamen right					
Pearson’s r	0.058	0.565^a^	0.186	0.130	0.247
*p*-value	0.882	0.044	0.765	0.780	0.637
Nucleus caudatus right					
Pearson’s r	0.037	0.663^a^	0.214	0.187	0.684
*p*-value	0.952	0.013	0.730	0.688	0.134
Putamen left					
Pearson’s r	–0.183	0.583^a^	0.302	–0.163	0.620
*p*-value	0.637	0.037	0.621	0.727	0.189
Nucleus caudatus left					
Pearson’s r	–0.041	0.520	–0.317	–0.524	0.688
*p*-value	0.917	0.069	0.603	0.228	0.131

*AD, Alzheimer’s disease; MD, movement disorders; PD, Parkinson’s disease; LBD, Lewy body dementia; PSP + CBD, progressive supranuclear palsy + corticobasal degeneration. Significant results are written in red.*

*^a^p < 0.05.*

*^b^p < 0.01.*

*^c^p < 0.001.*

### Phosphorylated Neurofilament Heavy Chain Also Correlates Positively With Dopamine Transporter Single Photon Emission Computed Tomography Pathology

Every patient in the AD and MD cohort had pathologically high pNfH levels according to the cutoff levels as provided by the manufacturer. The lowest levels of pNfH could be observed in patients with AD (0.41 ± 0.18 ng/ml), followed by patients with LBD (0.45 ± 0.17 ng/ml) and PD (0.51 ± 0.39 ng/ml) (see Table 1 and Figure 1). The highest levels could be observed in patients with PSP and CBD (0.61 ± 0.76 ng/ml). The pNfH levels are moderately elevated in the MD cohort (0.50 ± 0.55 ng/ml).

There is no correlation between CSF pNfH levels and z-scores of each brain region in patients with AD (see Table 4 and Figure 2). In the combined group of nigrostriatal movement disorders, positive correlations are seen in between pNfH levels in CSF and putamen right (*r* = 0.383, *p* < 0.05) and putamen left (*r* = 0.384, *p* < 0.05). Also, the CSF levels and DaTscan pathology correlated in the PSP and CBD cohort (putamen right *r* = 0.575, *p* < 0.05 and putamen left *r* = 0.571, *p* < 0.05).

**TABLE 4:** Pearson’s correlation phosphorylated neurofilament heavy chain (pNfH).

**Pearson’s correlation**	**AD**	**MD**	**PD**	**LBD**	**PSP + CBD**
Putamen right					
Pearson’s r	6.473*e*^−10^	0.438^a^	0.520	–0.319	0.575^a^
*p*-value	0.998	0.017	0.151	0.485	0.040
Nucleus caudatus right					
Pearson’s r	–0.140	0.308	0.299	–0.137	0.464
*p*-value	0.592	0.104	0.434	0.770	0.110
Putamen left					
Pearson’s r	0.021	0.397^a^	0.438	–0.034	0.571^a^
*p*-value	0.973	0.033	0.238	0.943	0.042
Nucleus caudatus left					
Pearson’s r	–0.085	0.362	0.403	0.251	0.417
*p*-value	0.746	0.054	0.282	0.587	0.156

*AD, Alzheimer’s disease; MD, movement disorders; PD, Parkinson’s disease; LBD, Lewy body dementia; PSP + CBD, progressive supranuclear palsy + corticobasal degeneration. Significant results are written in red.*

*^a^p < 0.05.*

*^b^p < 0.01.*

*^c^p < 0.001.*

### Alpha-Synuclein, Progranulin, and Total-Tau Show no Significant Correlation in Dopamine Transporter Single Photon Emission Computed Tomography and Cerebrospinal Fluid Levels

Comparing α-Syn levels of every subcohort with normal controls (mean level = 2,218.31 ± 888.19 pg/ml) revealed no significant deviation at all (see Table 4). The lowest levels could be observed in patients with PSP + CBD (1,854.2 ± 1,100.27 pg/ml). AD patients (2,012.85 ± 449.11 pg/ml) as well as patients with alpha-synucleinopathies had no significant deterioration of α-Syn levels (PD 1,885.16 ± 626.59 pg/ml; LBD 2,587.23 ± 1,339.17 pg/ml) (see Table 1). None of the subcohorts revealed a correlation between DaTscan and α-Syn CSF levels.

Progranulin levels in CSF were similar throughout the cohorts (AD 0.89 ± 0.18 pg/ml; MD 0.87 ± 0.25 pg/ml), no significant differences could be observed throughout the subgroups. In addition, we could not identify any significant correlations between CSF levels and dopamine transporter imaging.

Total tau is pathologically elevated in patients with AD (513.35 ± 240.76 pg/ml) with a cutoff at 450 (pg/ml) as given by the manufacturer. The second highest level can be found in patients with PSP and CBD (333.616 ± 320.77 pg/ml). There are no significant differences in T-tau in between each cohort or subgroup (see Table 2). T-tau did also not correlate with DaTscan z-scores.

## Discussion

We observed a strong correlation of CSF neurofilament levels with a pathological functional integrity of presynaptic dopamine neurons in patients with nigrostriatal degeneration across the different pathomechanisms. We report significant correlations for NfL for most striatal regions on both hemispheres. Another positive correlation could be observed between CSF pNfH levels and the left and right putamen in movement disorders.

A positive correlation of early neuronal damage in the nucleus caudatus and higher NfL levels in CSF have already been described but not for the putamen (Bäckström et al., 2020). One major role of the putamen is supporting the execution of the intended motorical plans (Vicente et al., 2012). The putamen has been shown as one of the first important brain locations to be affected in MD patients by a decreased dopaminergic metabolism (Kordower et al., 2013). Our results showing the predominance of the correlation with z-scores of the putamen are well in line with the pathophysiology since all of our patients received their spinal tap and DaTscan at the beginning of the disease. Earlier studies in PD showed that denervation of dopaminergic neurons in the putamen precedes denervation in the nucleus caudatus (Brooks and Piccini, 2006; Jakobson et al., 2013). Also, NfL has been shown as an early predictor of motor impairment in PD (Bäckström et al., 2020). Taken together, these two factors may explain the significant correlation as found here, reflecting the assumed pathophysiology and clinical course of diseases within nigrostriatal neurodegeneration. Also, both biomarkers, DaTscan and neurofilaments, reflect the underlying pathogenetic neurodegeneration. In general, the lowest NfL levels could be found in patients with PD and AD, the highest levels could be observed in patients with PSP and CBD. Pathologically high CSF NfL levels were only found in the PSP and CBD group. These elevated levels are in agreement with other studies (Hall et al., 2012; Magdalinou et al., 2015; Olsson et al., 2019). CSF levels for pNfH are also elevated in all groups in a similar manner. Thus, pNfH has a similar correlation also indicating direct or indirect involvement of neurofilaments in the loss of the presynaptic dopamine transporters. On the contrary, NfL CSF levels are not elevated in PD in general (Bridel et al., 2019; Bäckström et al., 2020). Neurofilament light chain is a well-known marker for axonal–neuronal degeneration with, on the one hand, low specificity for the underlying pathomechanisms except for Tar-DNA-binding protein with 43 kDa induced neurodegeneration such as in ALS (Weydt et al., 2016; Goossens et al., 2018; Körtvelyessy et al., 2018; Bridel et al., 2019). On the other hand, NfL levels do correlate with the speed of neurodegeneration as seen in ALS (Steinacker et al., 2016; Feneberg et al., 2018) and, e.g., Creutzfeld–Jacob disease (Palermo et al., 2020) meaning that high NfL levels reflect a fast disease progression. It is intriguing to speculate whether NfL is more specific for a loss of trajectories in between the striatal regions than just a neuronal degeneration. More studies are recommended to further investigate this hypothesis.

Progranulin has been of some interest in PD and related disorders because of its role in lysosomalen degeneration and in microglial activity (Tayebi et al., 2020). Progranulin levels were similar across every cohort and subgroup and did not differ from an age-related control cohort. Thus, PGRN levels do reflect the PGRN metabolism as it has been shown for patients with frontotemporal dementia with and without *GRN* mutations (Körtvélyessy et al., 2015; Wilke et al., 2017; Goossens et al., 2018; Körtvelyessy et al., 2018). In analogy, we could not see any change in the PGRN metabolism as mirrored in the CSF PGRN concentration in the entire movement disorder cohort.

Again, alpha-synuclein in CSF measured with our ELISA has not proven its biomarker properties as it has been several times before and is reviewed elsewhere (Mollenhauer and Trenkwalder, 2009; Fayyad et al., 2019).

Total-tau is a well-known marker for general neurodegeneration announced as one of the key biomarkers in AD (Jack et al., 2018). Here, this biomarker did not mirror the nigrostriatal neurodegeneration going on in our MD patients. This probably emphasizes the putative involvement of neurofilament and not general neuronal degeneration in the loss of presynaptic dopaminergic neurodegeneration.

This analysis has a number of limitations that should be acknowledged. One limitation is the small sample size of the groups, which leads to a higher risk of false-positive statistical test results, which could not be cross-checked in an independent cohort. Due to the fact that this study was done at the Department of Neurology and not at a movement disorder outpatient clinic, cohort and subgroup distributions are different with PSP + CBS being as frequent as PD, which is not a normal PD/PSP + CBD ratio for a movement disorder outpatient clinic or any neurological outpatient clinic.

## Conclusion

Neurofilament light chain and pNfH concentrations in the CSF are probably reflecting the specific loss of presynaptic dopamine transporter loss in the putamen only in patients with nigrostriatal neurodegeneration and concomitant movement disorders. We could also think of the DaTscan and NfL, pNfH levels reflecting two sides of the pathomechanisms.

We encourage further studies to correlate dopaminergic or amyloid imaging with fluid biomarkers such as YKL-40 (Baldacci et al., 2019) or neurogranin (Mazzucchi et al., 2020) to elucidate the systemic effect of neurodegeneration.

## Data Availability Statement

The original contributions presented in the study are included in the article/supplementary material, further inquiries can be directed to the corresponding author/s.

## Ethics Statement

The studies involving human participants were reviewed and approved by the Ethikkommission der Universitätsklinik Magdeburg. Written informed consent for participation was not required for this study in accordance with the national legislation and the institutional requirements.

## Author Contributions

PK, EDi, PG, and EDü: study concept and design. PK and EDi: writing the first draft and statistics. PK, EDi, IG, and SV: clinical data acquisition. All authors: writing and revising. All authors contributed to the article and approved the submitted version.

## Conflict of Interest

The authors declare that the research was conducted in the absence of any commercial or financial relationships that could be construed as a potential conflict of interest.

## Publisher’s Note

All claims expressed in this article are solely those of the authors and do not necessarily represent those of their affiliated organizations, or those of the publisher, the editors and the reviewers. Any product that may be evaluated in this article, or claim that may be made by its manufacturer, is not guaranteed or endorsed by the publisher.
